# Macrophages M1-Related Prognostic Signature in Hepatocellular Carcinoma

**DOI:** 10.1155/2021/6347592

**Published:** 2021-09-23

**Authors:** Hao Zhang, Lin Sun, Xiao Hu

**Affiliations:** ^1^Department of Hepatobiliary Pancreatic Surgery, The Affiliated Hospital of Qingdao University, Qingdao, Shandong, China; ^2^Department of ICU, The Affiliated Hospital of Qingdao University, Qingdao, Shandong, China

## Abstract

A large number of studies have found that macrophages M1 play an important role in the occurrence and development of tumors. The aim of our study is to explore the causes of differential infiltration of macrophages M1 in hepatocellular carcinoma from the perspective of transcriptome and establish a prognostic model of hepatocellular carcinoma. We downloaded gene expression and clinical data from the public database, estimated the content of macrophages M1 in different samples with R software, and found the different genes between high- and low-infiltration groups. Using differentially expressed genes, we constructed a model composed of 7 genes. The risk score of the model has a good ability to predict the prognosis, has a positive correlation with immune checkpoints, and is closely related to other immune cells and immune function. Our model shows good prognostic function and has wide application value.

## 1. Introduction

Liver cancer is a kind of malignant tumor disease with high incidence all over the world, which seriously endangers public health. Improving the prognosis of patients with liver cancer and curing liver cancer is one of the goals of researchers. The impact of the tumor immune microenvironment on liver cancer cells has been found to be more and more important. At present, there are a large number of studies on tumor immune microenvironment. Tumor-associated macrophages are a key factor in cancer. Macrophages play an important role in the development of tumors. They can promote genomic instability, promote the growth of tumor stem cells, promote metastasis, and so on [1]. Rodell et al. found that TLR7/8-agonist-loaded nanoparticles enhance cancer immunotherapy by macrophages M1 [2]. Chen et al. found that tumor-recruited M2 macrophages promote gastric and breast cancer metastasis [3]. Choo et al. found that M1 macrophage-derived nanovesicles potentiate the anticancer efficacy of immune checkpoint inhibitors [4]. Rao et al. found that hybrid cellular membrane nanovesicles amplify macrophage immune responses against cancer recurrence and metastasis [5].

At present, a considerable number of studies have found that some genes can affect the prognosis of cancer patients. Conlin et al. found that K-ras, p53, and APC mutations had prognostic significance in colorectal carcinoma [6]. Powell et al. found that p53 is a prognostic significance in breast cancer [7]. Gurung et al. found that AIMP3 predicts survival following radiotherapy in muscle-invasive bladder cancer [8]. In recent years, a large number of models were constructed by multiple genes that can accurately predict the prognosis of patients. Deng et al. found that a five-autophagy-related lncRNA signature was used to be a prognostic model in HCC [9]. Feng et al. found a 7-gene prognostic signature to predict the survival of pancreatic ductal adenocarcinoma [10]. Yin et al. found a novel prognostic six-CpG signature in glioblastomas [11].

The aim of our study is to explore the causes of differential infiltration of macrophages M1 in hepatocellular carcinoma from the perspective of transcriptome. Using differentially expressed genes to construct a reliable prognosis model is expected to improve the prognosis of patients with HCC. In our model, we scored the content of macrophages M1 according to the transcriptome data downloaded from The Cancer Genome Atlas and found the differentially expressed genes between high- and low-infiltration groups. The prognostic model was constructed according to the differential genes and verified on the external database. Our model is also deeply discussed.

## 2. Materials and Methods

### 2.1. Data Download

We downloaded the expression data of the hepatocellular liver carcinoma project rectified to fragments perkilobase million (FPKM) as the training cohort and clinical data of HCC in The Cancer Genome Atlas (TCGA, https://tcga-data.nci.nih.gov/tcga/). The expression data and clinical data of Liver Cancer-RIKEN, Japan, were downloaded from the International Cancer Genome Consortium (ICGC, https://dcc.icgc.org/). We annotated the data by gene transfer format (GTF) files obtained from Ensembl (http://asia.ensembl.org).

### 2.2. Construction and Validation of the Model

Screening of DEGs was carried out by “limma” package (https://bioconductor.org/packages/limma/) in R software (4.0.0). The data were analyzed by Cox hazard analysis and Lasso regression with the “survival” (https://cran.r-project.org/package=survival), “glmnet” (https://cran.r-project.org/package=glmnet), and “survminer” (https://cran.r-project.org/package=survminer) package. The “survivalROC” package was used to draw receiver operating characteristic curve, and the “survival” package was used to draw the survival curve.

### 2.3. Gene Set Enrichment Analysis (GSEA)

GSEA was utilized in this study to find the differences between different risk groups in the TCGA cohort. An annotated gene set file (c2.cp.kegg.v7.0.symbols.gmt) was selected as the reference. The threshold was confirmed as FDR q-val <0.05.

### 2.4. The Analysis of Immune

Significant results of immune infiltrate deconvolution were obtained in TCGA patients with HCC by CIBERSORT analysis. The “StromalScore,” “ImmuneScore,” and “ESTIMATEScore” of each sample in the TCGA cohort are carried out by the “estimate” package. The “GSVA” and “GSEABase” packages were used for ssGSEA analysis for each patient. The correlation analysis of each index was completed by the Spearman test.

## 3. Result

### 3.1. Constructing the Prognosis Model in the TCGA Cohort

**Figure 1:**
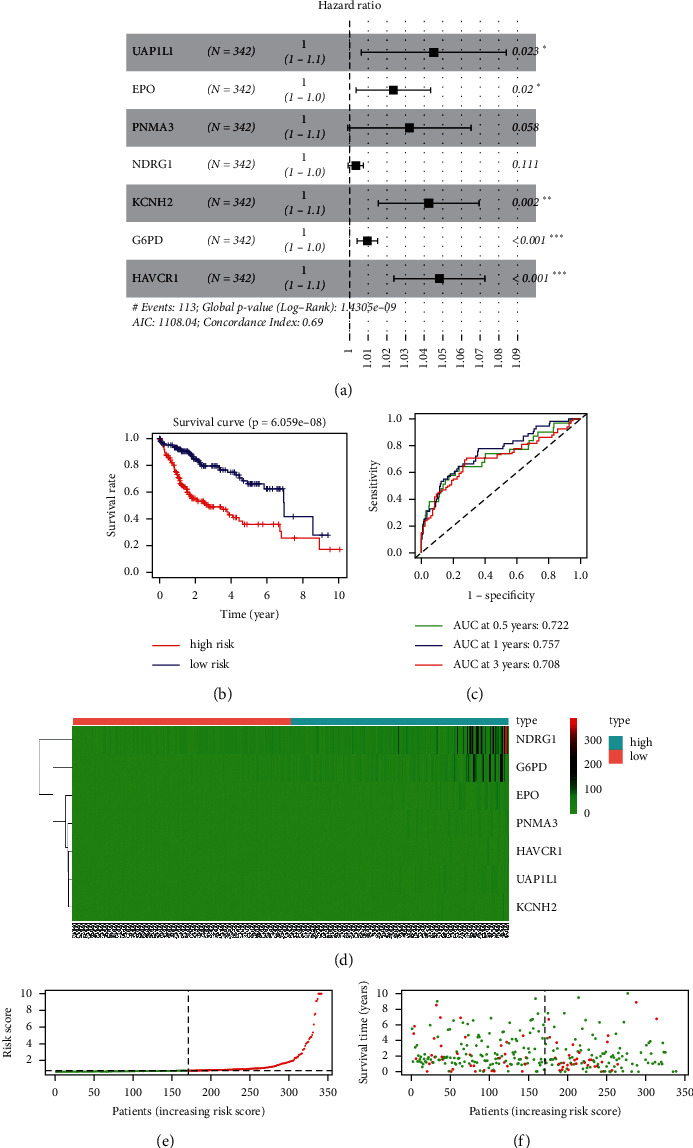
Constructing the prognosis model. (a) The result of multivariate Cox hazard analysis. (b) Comparison of survival status between the high-risk group and low-risk group. (c) The ROC curves at different years in the TCGA cohort. (d) The expression level of 7 genes in different groups. ((e) and (f)) The survival sates of different risk score patients.

After scoring the macrophages M1 of different HCC patients, we ranked the scores from low to high. Analyzing the DEGs between the first quarter of patients (86) and the last quarter of patients (87), 317 DEGs were found in the process. Combined with the clinical prognosis, we screened 55 genes by univariate Cox hazard analysis in the TCGA cohort. We used Lasso regression and multivariate Cox hazard analysis to narrow the number of genes and finally got 7 genes to optimize the model (Figure 1(a)), and the risk score of each sample was calculated (risk score = UAP1L1 ∗ 0.0433 + EPO ∗ 0.0226 + PNMA3 ∗ 0.0307 + NDRG1 ∗ 0.0032 + KCNH2 ∗ 0.0406 + G6PD ∗ 0.0092 + HAVCR1 ∗ 0.0460) and the median of risk score was used to distinguish the high- and low-risk group. In the 0.5, 1, and 3 years, the AUC value under the ROC curve is 0.722, 0.757, and 0.708 (Figure 1(b)). There were significant differences in prognosis between high- and low-risk groups (Figure 1(c)). The heatmap showed that the expression level of UAP1L1, EPO, PNMA3, NDRG1, KCNH2, G6PD, and HAVCR1 in the high-risk group was higher than that in the low-risk group (Figure 1(d)) and the risk of death in HCC patients increased with the increase in risk score (Figures 1(e) and 1(f)).

### 3.2. Verifying the Prognosis Model

**Figure 2:**
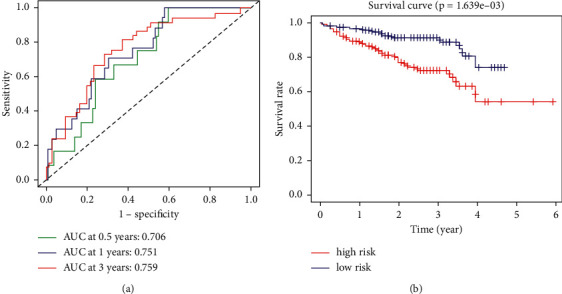
Verifying the prognosis model. (a) The ROC curves at different years in the ICGC cohort. (b) Comparison of survival status between different groups in the ICGC cohort.

We validated the model in the GSE14520 cohort. In the 0.5, 1, and 3 years, the AUC values under the ROC curve are 0.706, 0.751, and 0.759 (Figure 2(a)). The model can significantly distinguish the prognosis of patients in high- and low-risk groups (Figure 2(b)).

### 3.3. The Risk Score Was an Independent Prognostic Indicator

**Figure 3:**
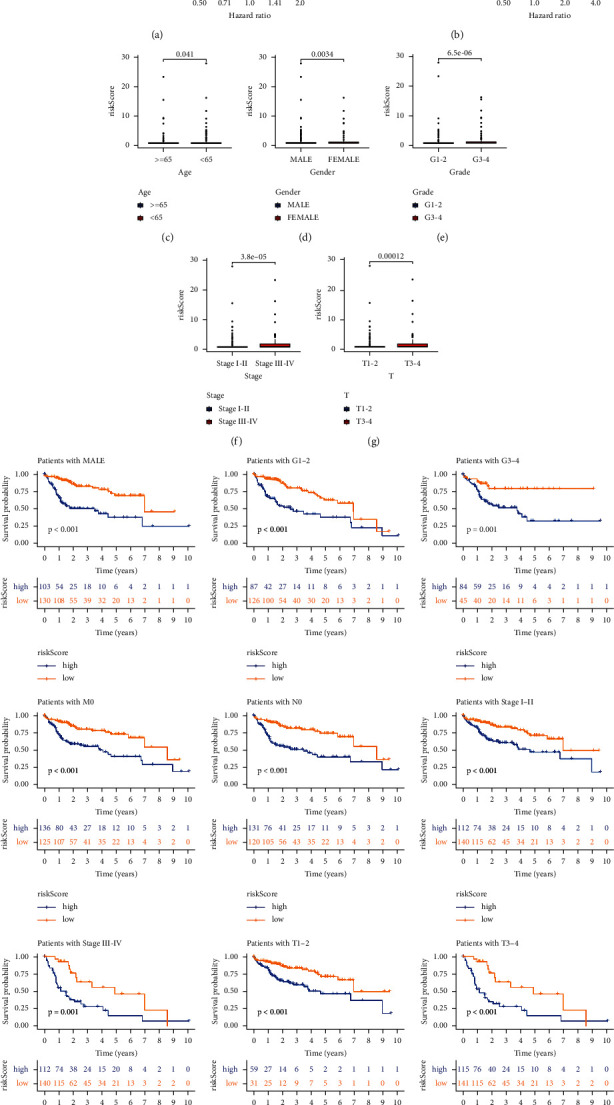
Relationship between risk score and clinicopathological features. Univariate Cox hazard analysis (a) and multivariate Cox hazard analysis (b) of patient's features. The distribution of risk score in different ages (c), genders (d), grades (e), stages (f), and *T* (g) has a significant difference. (h) The risk score can predict the survival of patients in different age, gender, histological grade, M0, N0, stage, and *T* patients.

We analyzed the relationship between risk score and clinicopathological characteristics (age, gender, histological grade, clinical stage, and TNM). Univariate Cox hazard analysis of clinicopathological features showed that the *p* value of stage, *T,* and risk score was less than 0.001 and the hazard ratio was over 1 (Figure 3(a)). Multivariate Cox hazard analysis of clinicopathological features showed that the *p* value of risk score was less than 0.05 and the hazard ratio was over 1 (Figure 3(b)). The risk score in different ages, genders, grades, stages, and *T* groups has significant differences (Figures 3(c)–3(f)). There are significant differences in the prognosis of different risk score groups in different ages, genders, histological grades, M0, N0, stages, and *T* (Figure 3(g)).

### 3.4. The GSEA of Different Risk Score Groups

**Figure 4:**
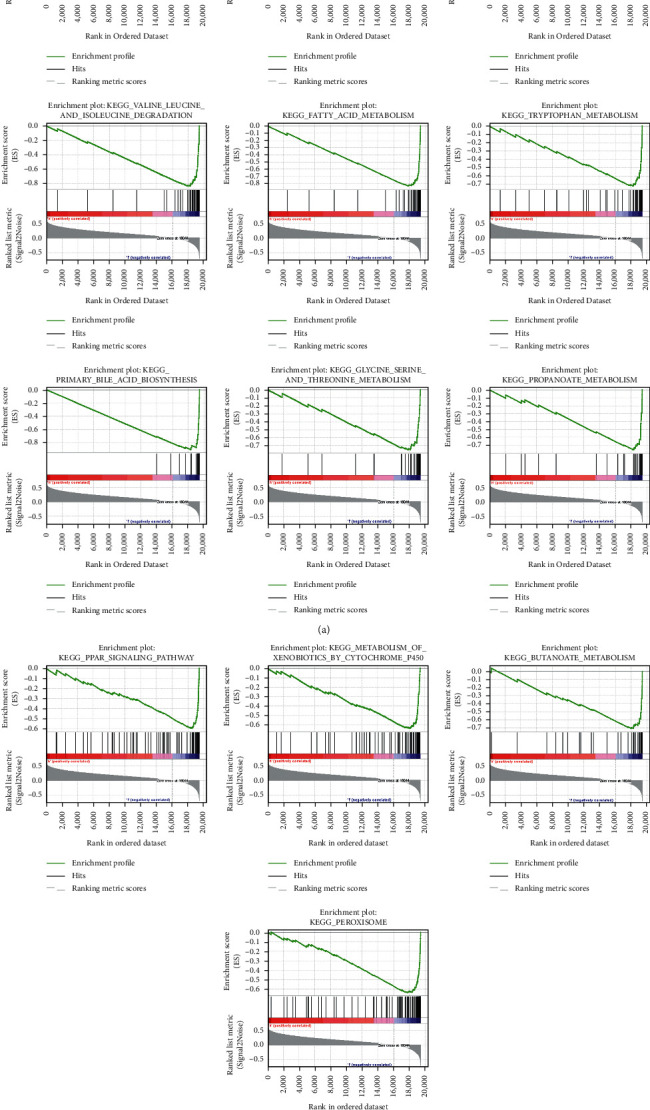
The gene sets of the low-risk group.

In the high-risk group, 0 gene sets were found (FDR q-val <0.05). In the low-risk group, we found 18 gene sets, including DRUG_METABOLISM_CYTOCHROME_P450, COMPLEMENT_AND_COAGULATION_CASCADES, RETINOL_METABOLISM, VALINE_LEUCINE_AND_ISOLEUCINE_DEGRADATION, FATTY_ACID_METABOLISM, TRYPTOPHAN_METABOLISM, PRIMARY_BILE_ACID_BIOSYNTHESIS, GLYCINE_SERINE_AND_THREONINE_METABOLISM, PROPANOATE_METABOLISM, PPAR_SIGNALING_PATHWAY, METABOLISM_OF_XENOBIOTICS_BY_CYTOCHROME_P450, and BUTANOATE_METABOLISM (Figure 4) (FDR q-val <0.001).

### 3.5. The Risk Score and Immune

**Figure 5:**
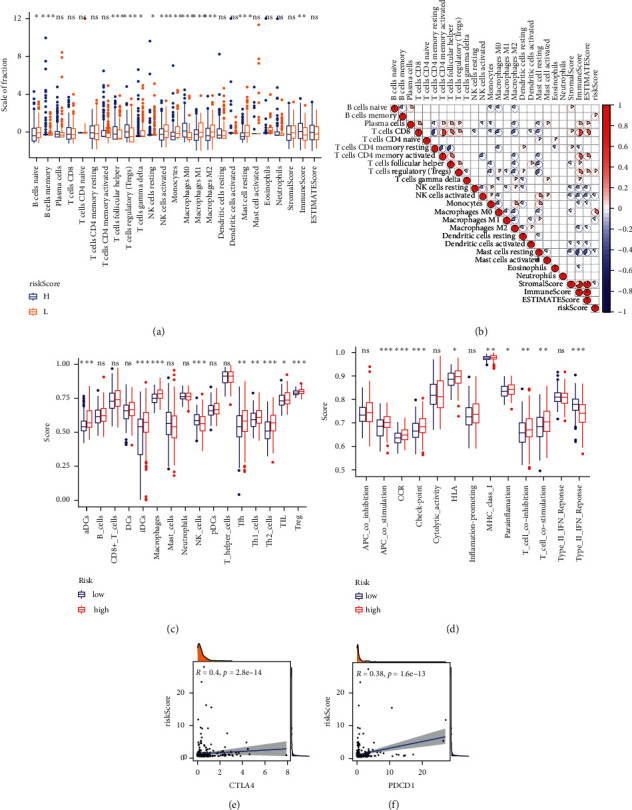
The risk score and immune. (a) Differences of immune cells among different risk score groups. (b) Correlation between immune cells and risk score. (c, d) The ssGSEA analysis of immune cells and immune function in different risk score groups. (e, f) The correlation between risk score and immune checkpoint.

We found that the content of macrophages M1 can be well distinguished among different risk score groups. There were also significant differences in the content of some immune cells in different risk score groups (Figure 5(a)). There was a significant correlation between risk score and macrophages M1 (Figure 5(b)). The ssGSEA analysis showed that there was no significant difference in the cell content of B cells, CD8 *T* cells, DCs, mast_cells, neutrophils, pDCs, and *T* helper cells in the high- and low-risk groups and APC coinhibition, cytolytic activity, inflammation promoting, and type 1 INF reponse (Figures 5(c) and 5(d)). We also found a significant positive correlation between risk score and immune checkpoint (CTLA4 and PDCD1) (Figure 5(e)).

## 4. Discussion

Macrophages M1 in hepatocellular carcinoma have been concerned by a large number of researchers. During the differentiation of monocytes into macrophages, macrophages obtain immunosuppressive function in order to maintain the homeostasis of the immune microenvironment, but the M1 polarization of macrophages has a significant antitumor effect [12]. Macrophages secrete vascular endothelial growth factor, platelet-derived growth factor, and transforming growth factor *β* which inhibited antitumor immunity and promoted tumor progression [13–15]. These findings also provide a new dimension for the immunosuppressive effect of cancer. Angiogenesis inhibition therapy has also become a promising treatment strategy for HCC [16]. Zhao et al. found that the miR-144/miR-451a cluster could promote macrophage M1 polarization and antitumor activity in HCC [17]. Sprinzl et al. found that macrophage might contribute to the anticancer activity of sorafenib [18]. Kim et al. found that hippo signaling suppresses macrophage infiltration in HCCs [19]. Clinical trials that exert influence on macrophages have shown improvement on tumors. The prognostic significance of combining tumor-secreted osteopontin with microenvironment-associated peritumoral macrophages was confirmed in HCC with early stage [20]. The combination of bavituximab with sorafenib could increase the frequency of M1 macrophages in the treatment for advanced HCC patients [21]. Terakawa et al. found that the capability of macrophages to produce TNF-alpha could be useful for prognostis and for monitoring immunocompetence in patients with pancreatic cancer [22].

The immune microenvironment of HCC is quite complex. In particular, the relationship between macrophages and Tregs has been widely concerned. Macrophages aggregate Tregs cells to cancer sites by expressing CCL17, CCL18, and CCL22, thus hindering the activation of cytotoxic *T* cells [23, 24]. Granito et al. [25] found that tumor-associated macrophages (by secreting IL-10) can induce CD4+ CD25+ Foxp3 regulatory *T* cells, thus indirectly supporting tumor growth and progression. It was found that the IL-10 antibody could partially block the aggregation effect of macrophages on Tregs [26].

The genes in our model play an important role in tumors. Hill et al. identified UAP1L1 is a methylated gene associated with clinical features [27]. Lai et al. found that UAP1L1 is a critical factor for protein O-GlcNAcylation and cell proliferation in human hepatoma cells [28]. Bradbury found that EPO helps children with cancer-related anaemia [29]. Kumar et al. found that EPO receptor contributes to melanoma cell survival [30]. Schüller et al. found that PNMA3 is a novel neuronal protein implicated in paraneoplastic neurological disease [31]. Sevinsky et al. found that NDRG1 regulates neutral lipid metabolism in breast cancer [32]. Villodre et al. found that NDRG1 is an independent prognostic factor in breast cancer [33]. Afrasiabi et al. found that KCNH2 regulated melanoma cell proliferation and migration [34]. Feng et al. found that G6PD regulated paclitaxel resistance in ovarian cancer [35]. Liu et al. found that HAVCR1 might be a novel prognostic factor for gastric cancer [36].

There is a significant correlation between the risk score in our model and many immune indexes and immune checkpoints, which is a very meaningful discovery. However, our model needs more biological function verification and multicenter patient data to modify our model. It is hoped that our model can provide new ideas for the treatment of hepatocellular carcinoma and improve the prognosis of hepatocellular carcinoma patients.

List of abbreviations: HCC, hepatocellular carcinoma; FPKM, fragments perkilobase million; TCGA, The Cancer Genome Atlas; GTF, gene transfer format; DEGs, differentially expressed genes; GSEA, gene set enrichment analysis.

## Data Availability

The datasets used and/or analyzed during the current study are available from the corresponding author on reasonable request.

## References

[1] Mantovani A., Marchesi F., Malesci A., Laghi L., Allavena P. (2017). Tumour-associated macrophages as treatment targets in oncology. *Nature Reviews Clinical Oncology*.

[2] Rodell C. B., Arlauckas S. P., Cuccarese M. F. (2018). TLR7/8-agonist-loaded nanoparticles promote the polarization of tumour-associated macrophages to enhance cancer immunotherapy. *Nature Biomedical Engineering*.

[3] Chen Y., Zhang S., Wang Q., Zhang X. (2017). Tumor-recruited M2 macrophages promote gastric and breast cancer metastasis via M2 macrophage-secreted CHI3L1 protein. *Journal of Hematology & Oncology*.

[4] Choo Y. W., Kang M., Kim H. Y. (2018). M1 macrophage-derived nanovesicles potentiate the anticancer efficacy of immune checkpoint inhibitors. *ACS Nano*.

[5] Rao L., Wu L., Liu Z. (2020). Hybrid cellular membrane nanovesicles amplify macrophage immune responses against cancer recurrence and metastasis. *Nature Communications*.

[6] Conlin A., Smith G., Carey F. A., Wolf C. R., Steele R. J. (2005). The prognostic significance of K-ras, p53, and APC mutations in colorectal carcinoma. *Gut*.

[7] Powell B., Soong R., Iacopetta B., Seshadri R., Smith D. R. (2000). Prognostic significance of mutations to different structural and functional regions of the p53 gene in breast cancer. *Clinical Cancer Research: An Official Journal of the American Association for Cancer Research*.

[8] Gurung P. M., Veerakumarasivam A., Williamson M. (2015). Loss of expression of the tumour suppressor gene AIMP3 predicts survival following radiotherapy in muscle-invasive bladder cancer. *International Journal of Cancer*.

[9] Deng X., Bi Q., Chen S. (2020). Identification of a five-autophagy-related-lncRNA signature as a novel prognostic biomarker for hepatocellular carcinoma. *Frontiers in Molecular Biosciences*.

[10] Feng Z., Qian H., Li K., Lou J., Wu Y., Peng C. (2021). Development and validation of a 7-gene prognostic signature to improve survival prediction in pancreatic ductal adenocarcinoma. *Frontiers in Molecular Biosciences*.

[11] Yin A.-A., Lu N., Etcheverry A. (2018). A novel prognostic six-CpG signature in glioblastomas. *CNS Neuroscience and Therapeutics*.

[12] Lu C., Rong D., Zhang B. (2019). Current perspectives on the immunosuppressive tumor microenvironment in hepatocellular carcinoma: challenges and opportunities. *Molecular Cancer*.

[13] Zhang D., Qiu X., Li J., Zheng S., Li L., Zhao H. (2018). TGF-*β* secreted by tumor-associated macrophages promotes proliferation and invasion of colorectal cancer via miR-34a-VEGF axis. *Cell Cycle*.

[14] Darvishi B., Majidzadeh A. K., Ghadirian R., Mosayebzadeh M., Farahmand L. (2019). Recruited bone marrow derived cells, local stromal cells and IL-17 at the front line of resistance development to anti-VEGF targeted therapies. *Life Sciences*.

[15] Deryugina E. I., Quigley J. P. (2015). Tumor angiogenesis: MMP-mediated induction of intravasation-and metastasis-sustaining neovasculature. *Matrix Biology*.

[16] Liu J.-Y., Chiang T., Liu C.-H. (2015). Delivery of siRNA using CXCR4-targeted nanoparticles modulates tumor microenvironment and achieves a potent antitumor response in liver cancer. *Molecular Therapy*.

[17] Zhao J., Li H., Zhao S. (2021). Epigenetic silencing of miR-144/451a cluster contributes to HCC progression via paracrine HGF/MIF-mediated TAM remodeling. *Molecular Cancer*.

[18] Sprinzl M. F., Puschnik A., Schlitter A. M. (2015). Sorafenib inhibits macrophage-induced growth of hepatoma cells by interference with insulin-like growth factor-1 secretion. *Journal of Hepatology*.

[19] Kim W., Khan S. K., Liu Y. (2018). Hepatic hippo signaling inhibits protumoural microenvironment to suppress hepatocellular carcinoma. *Gut*.

[20] Zhu W., Guo L., Zhang B. (2014). Combination of osteopontin with peritumoral infiltrating macrophages is associated with poor prognosis of early-stage hepatocellular carcinoma after curative resection. *Annals of Surgical Oncology*.

[21] Cheng X., Li L., Thorpe P. E., Yopp A. C., Brekken R. A., Huang X. (2016). Antibody-mediated blockade of phosphatidylserine enhances the antitumor effect of sorafenib in hepatocellular carcinomas xenografts. *Annals of Surgical Oncology*.

[22] Terakawa N., Satoi S., Takai S. (2006). Clinical monitoring of innate cellular immunity of monocytes/macrophages by tumor necrosis factor alpha productivity in whole blood stimulated by lipopolysaccharide in patients with pancreatic cancer. *Pancreas*.

[23] Wang D., Yang L., Yue D. (2019). Macrophage-derived CCL22 promotes an immunosuppressive tumor microenvironment via IL-8 in malignant pleural effusion. *Cancer Letters*.

[24] Mamrot J., Balachandran S., Steele E. J., Lindley R. A. (2019). Molecular model linking Th2 polarized M2 tumour‐associated macrophages with deaminase‐mediated cancer progression mutation signatures. *Scandinavian Journal of Immunology*.

[25] Granito A., Muratori L., Lalanne C. (2021). Hepatocellular carcinoma in viral and autoimmune liver diseases: role of CD4+ CD25+ Foxp3+ regulatory *T* cells in the immune microenvironment. *World Journal of Gastroenterology*.

[26] Zhou J., Ding T., Pan W., Zhu L.-Y., Li L., Zheng L. (2009). Increased intratumoral regulatory *T* cells are related to intratumoral macrophages and poor prognosis in hepatocellular carcinoma patients. *International Journal of Cancer*.

[27] Hill V. K., Ricketts C., Bieche I. (2011). Genome-wide DNA methylation profiling of CpG islands in breast cancer identifies novel genes associated with tumorigenicity. *Cancer Research*.

[28] Lai C.-Y., Liu H., Tin K. X. (2019). Identification of UAP1L1 as a critical factor for protein O-GlcNAcylation and cell proliferation in human hepatoma cells. *Oncogene*.

[29] Bradbury J. (2006). Erythropoietin helps children with cancer-related anaemia. *The Lancet Oncology*.

[30] Kumar S. M., Zhang G., Bastian B. C. (2012). Erythropoietin receptor contributes to melanoma cell survival in vivo. *Oncogene*.

[31] Schüller M., Jenne D., Voltz R. (2005). The human PNMA family: novel neuronal proteins implicated in paraneoplastic neurological disease. *Journal of Neuroimmunology*.

[32] Sevinsky C. J., Khan F., Kokabee L., Darehshouri A., Maddipati K. R., Conklin D. S. (2018). NDRG1 regulates neutral lipid metabolism in breast cancer cells. *Breast Cancer Research*.

[33] Villodre E. S., Gong Y., Hu X. (2020). NDRG1 expression is an independent prognostic factor in inflammatory breast cancer. *Cancers*.

[34] Afrasiabi E., Hietamäki M., Viitanen T., Sukumaran P., Bergelin N., Törnquist K. (2010). Expression and significance of HERG (KCNH_2_) potassium channels in the regulation of MDA-MB-435S melanoma cell proliferation and migration. *Cellular Signalling*.

[35] Feng Q., Li X., Sun W. (2020). Targeting G6PD reverses paclitaxel resistance in ovarian cancer by suppressing GSTP1. *Biochemical Pharmacology*.

[36] Liu L., Song Z., Zhao Y. (2018). HAVCR1 expression might be a novel prognostic factor for gastric cancer. *PLoS One*.

